# A comprehensive dataset on the effects of *Nannochloropsis* sp. inclusion diets on water quality and oxidative stress of guppy (*Poecilia reticulata*)

**DOI:** 10.1016/j.dib.2022.108820

**Published:** 2022-12-13

**Authors:** Razia Sultana, Helena Khatoon, Mohammad Redwanur Rahman, Mohammad Ekramul Haque, Md. Sajjadul Mustaquim, Zannatul Nayma, Fardous Ara Mukta

**Affiliations:** Department of Aquaculture, Chattogram Veterinary and Animal Sciences University, Chattogram 4225, Bangladesh

**Keywords:** Guppy, *Nannochloropsis* sp., Water quality, Carotenoid, Oxidative stress

## Abstract

This experiment was designed to collect the data on antioxidants content of guppy fed with *Nannochloropsis* sp. inclusion diets through partial replacement of fishmeal in feed and the effect of microalgal diet on water quality parameter of the culture system. Triplicate groups of fifteen uniform sized guppy fries were kept in each rectangular glass tank (20 L) maintaining the male and female ratio to 1:2. Different experimental diets containing *Nannochloropsis* sp. (0%-control; 5%-N5, 10%-N10 and 15%-N15) and commercial feed (CMF) were fed to the fishes, two times a day at 5% of their body weight for 100 consecutive days. Water quality parameters were analyzed and recorded throughout the trial period. Both physical and chemical parameters of the culture tanks were measured during the trial period. At the end of experiment, random sampling was done for growth parameter assessment and further laboratory analysis. Oxidative stress (hydrogen peroxide and lipid peroxidation) was analyzed with the carcass sample. In this study, antioxidants content of guppy showed a significant difference among the treatment. Also, improved water quality parameters were found in the treatment tanks where guppy were fed with microalgae formulated feeds. In conclusion, results from this study indicate that selected marine microalga can increase the antioxidant properties of fish that would help in production of more hardy culture species for commercial aqua farming as well as help to maintain water quality parameters of the culture system which is now become a great problem.

**Specifications Table**

**Table:** 

Subject	Food Science, Aquatic Science
Specific subject area	Effect of microalgae formulated feed on survival, water quality and antioxidants
Type of data	Graph and Table
How the data were acquired	Data on oxidative stress (hydrogen peroxide and lipid peroxidation) were attained by biochemical and photometric analysis. Data of physical parameters of culture water such as- dissolve oxygen, temperature, pH were collected with glass thermometer, a portable pH meter (pHep-HI98107, HANNA, Romania) and dissolved oxygen meter (DO-5509, Lutron). Chemical parameters (NO_2__—_N, TAN and SRP) were determined following the methods of Parsons et al. (1984) by using a spectrophotometer. The collected data were analyzed by using MS Excel and IBM SPSS (v. 26.0) software.
Data format	Raw and analyzed
Description of data collection	For oxidative stress: hydrogen peroxide and lipid peroxidation level For physical parameters of water quality: temperature, pH, dissolve oxygen For chemical parameters of water quality: Nitrite nitrogen, total ammonia nitrogen and soluble reactive phosphorus
Data source location	Institution: Chattogram Veterinary and Animal Sciences University City/Town/Region: Khulshi-4225, Chattogram Country: Bangladesh
Data accessibility	Repository name: Mendeley Data Data identification number: DOI:10.17632/xnpj6353yw.1 Direct URL to data: https://data.mendeley.com/datasets/xnpj6353yw/1
Related research article	R. Sultana, H. Khatoon, M.R. Rahman, M.E. Haque, Z. Nayma, F.A. Mukta. Potentiality of *Nannochloropsis* Sp. As Partial Dietary Replacement of Fishmeal on Growth, Proximate Composition, Pigment and Breeding Performance in Guppy (*Poecilia Reticulata*). Bioresource Technol. Rep. 18 (2022) 101,112.

## Value of the Data

• Data on water quality parameters confirms that microalgae can be utilized as a potential source to improve the water quality of the culture tanks without any negative effect on the growth performance of fish.
• The data on oxidative stress indices signify the value of microalgae on health and survival of fish that provides a base platform for the researchers to generate alternative way of fish culture for high production and quality improvement in fish farming.
• Results obtained from this study indicate that including microalgae as a feed ingredient can improve the water quality of commercial fish culture tank which will reduce the water treatment cost as well as the production of healthy fish will boost the production and profitability.

## 1 Data Description

**Fig. 1:**
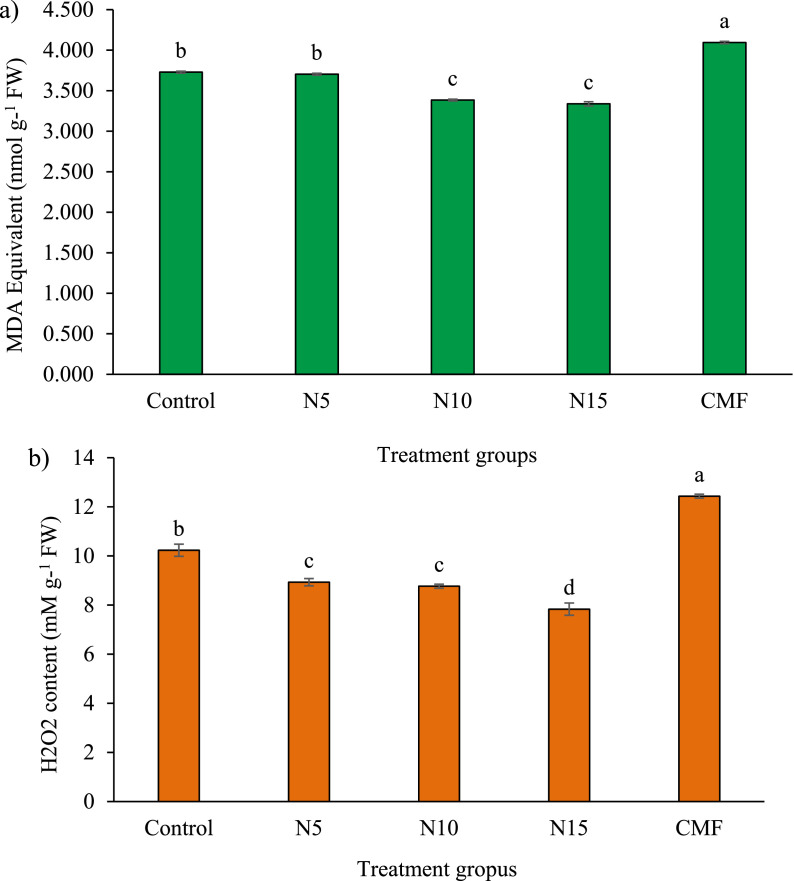
Level of lipid peroxidation (a) and hydrogen peroxide (H_2_O_2_) content (b) found in guppy fed with formulated diets after 100 consecutive days of culture period. Values are means ± standard error. Data were presented with different letters indicating significant (*P* < 0.05) differences.

**Fig. 2:**
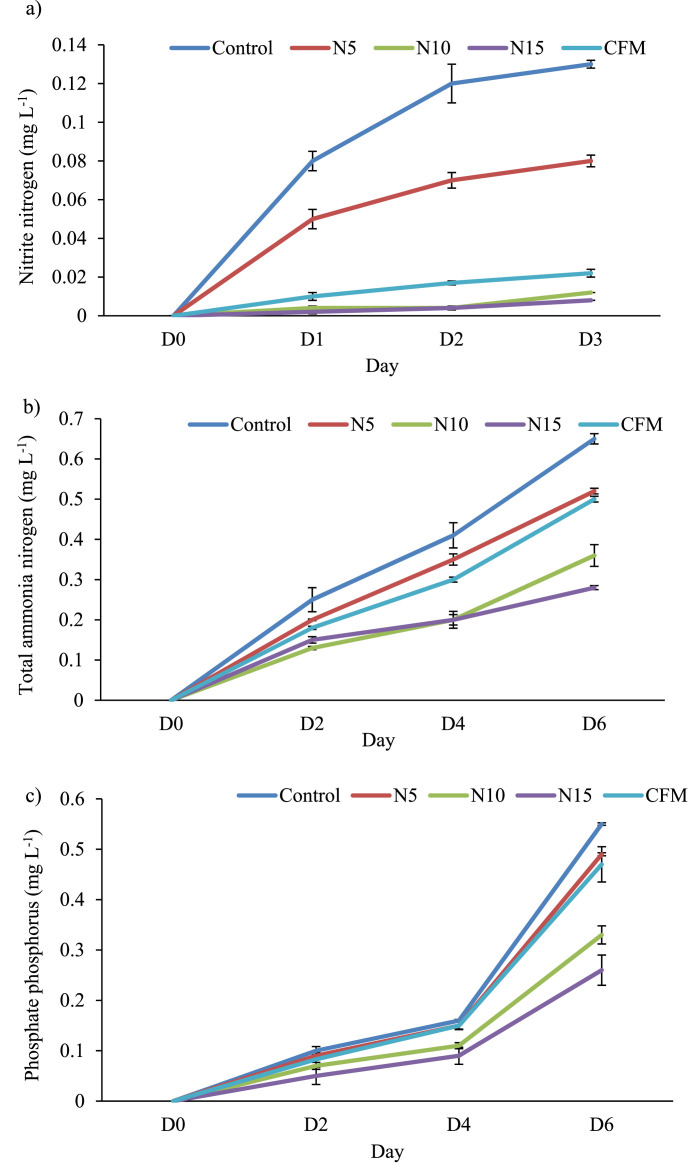
a. Nitrite nitrogen, b. total ammonia nitrogen, c. phosphate phosphorus concentration of the control and treatment tanks. Values are means ± standard deviation.

Fig. 1 showed the oxidative stress indices which included hydrogen peroxide (7.83–12.43 mM g-^1^) and lipid peroxidation (3.338 - 4.095 nmol g-^1^) in the fin and muscle of guppy at the termination of the experiment. Water quality was defined by physical parameters- dissolve oxygen (DO) (mg/L), temperature (°C) and pH (Table 1). Chemical parameters such as nitrite nitrogen, total ammonia nitrogen and soluble reactive phosphorus were varied among the different culture tanks which were represented in Fig. 2.

**Table 1:** Temperature, DO and pH of the control and treatment tanks. Values are means ± standard error. Different letters with the values indicate significant (*P* < 0.05) differences among the treatments.

Treatment	Parameter		
	Temperature ( °C)	DO (mg *L* ^−^ ^1^)	pH
Control	26.92 ± 0.06^a^	6.13 ± 0.23^a^	8.23 ± 0.03^a^
N5	26.95 ± 0.04^a^	6.03 ± 0.15^a^	8.29 ± 0.03^a^
N10	27.03 ± 0.08^a^	5.97 ± 0.18^a^	8.01 ± 0.09^a^
N15	26.98 ± 0.08^a^	6.23 ± 0.18^a^	7.90 ± 0.05^a^
CMF	27.10 ± 0.07^a^	6.00 ± 0.21^a^	7.97 ± 0.07^a^

## 2 Experimental Design, Materials and Methods

### 2.1 Culture of *Nannochloropsis* sp.

#### 2.1.1 Collection and Preparation of Pure Inoculums

Pure isolates of *Nannochloropsis* sp. were inoculated in the Conway medium using the formula described by Tompkins et al. (1995) [1] where 100 ml of pure *Nannochloropsis* sp. seed was inoculated in 900 ml of Conway medium to maintain the stock culture. Then the cultures were incubated in controlled indoor condition for 15 days at 24 °C temperature. Cool inflorescence lamps with 2000 lux light intensity were installed in the room to provide a 12 h light: 12 h dark regime [2].

#### 2.1.2 Mass Culture in Glass Tanks

Sea water was collected from Cox's Bazar to prepare media for mass culture. To eliminate debris, the collected sea water was first stored in plastic tanks overnight to allow the solid particles to settle down appropriately; filtered with a filter bag and vacuum filter pump to separate fine particles from the water and then sterilized in an autoclave (for 15 mins in 121 °C and 15 lbs). Then, the growth media was prepared with required nutrients; salinity and pH were adjusted to 26 ppt and 7.8, optimum for the culture of *Nannochloropsis* sp. [3]. Pure strains from the stock were transferred to 20 L glass tanks for mass culture at room temperature with a 12 h light: 12 h dark photoperiod, commencing with a volume of 5 L and subsequently scaling up to 16 L. Every two days, media was added to the culture tank until it reached the desired volume. Individual culture tanks were supplied with continuous aeration through a central air pump. Transparent polythene was used to cover the top sides of culture tanks to prevent contamination. Every week, samples from each tank were taken and examined under a light microscope for any signs of contamination. Temperature within the algal culture unit was measured daily.

#### 2.1.3 Harvest of Microalgae

A 16-day culture trial was performed to determine the native strain's stationary phase in a previous investigation [3] and according to this findings microalgae was cultured, harvested by centrifugation [4] and dried in a hot air oven at 60 °C temperature for overnight [5]. The dried biomass was pulverized using small sized mortar and pestle. The finely powdered algal biomass was then stored in airtight glass vials at 4 °C temperature until it could be used in the production of test diets.

### 2.2 Experimental Design

#### 2.2.1 Test Diet Preparation

All of the formulated test diets were approximately isoproteic and isolipidic (crude protein-41% and lipid-8%) (Table 2). The necessary feed ingredients such as, processed feed grade fishmeal, rice bran; wheat flour, vitamin + mineral premix, and commercial feed were purchased from local stores in Chattogram City (Bangladesh) (Table 3). First, fishmeal and rice bran were sieved and oven-dried at 60 °C to reduce the moisture level. Then they were crushed in the grinder to make more fine powder. To make dough, fishmeal, rice bran and wheat flour were mixed together and then *Nannochloropsis* sp. powder was added to that mixture as required. For comparative study, dried *Nannochloropsis* sp. powder was incorporated into the diet as follows: control (no inclusion), 5% (N5), 10% (N10), and 15% (N15). After mixing all of the ingredients together, an adequate amount of water was added to form a doughy texture. Then the doughy mixture was run through a grinder, dried, and granulated. The diets were sieved to ensure uniform granule size. Before final approval, all of the experimental diets as well as the ingredients used in diet preparation were subjected to chemical analysis. Crude protein (Kjeldahl Auto System) (ISO 5983–1987) and crude lipid (Soxtec HT6) of all the formulated feeds were determined to ensure homogenous nutritional quality. Finally, the prepared feeds were sealed in plastic bags, placed in airtight labeled containers, and stored in a cool (4 °C) dry place away from direct sunlight to preserve the feed's quality and prevent mold growth.

**Table 2:** Proximate composition of the harvested microalgae (*Nannochloropsis* sp.).

Nutritional Parameter	Percentage (%)
Crude Protein	32 ± 2.3% dry weight
Crude Lipid	26 ± 1.84% dry weight
Carbohydrate	29 ± 1.34% dry weight

**Table 3:** Percent ingredients and proximate composition of the experimental diets.

	Dietary group				
	Control	Microalgae diet			Commercial
		N5	N10	N15	CMF
Ingredients					
Fish meal	62%	60%	58%	56%	–
Rice bran	28%	25%	22%	19%	–
Wheat flour	8%	8%	8%	8%	–
vitamins + minerals	2%	2%	2%	2%	–
Microalgae powder	–	5%	10%	15%	–
Proximate composition					
Crude protein (%)	41.32%	41.29%	41.26%	41.23%	41.32%
Lipid (%)	8.14%	8.21%	8.23%	8.28%	7.9%

#### 2.2.2 Collection and Stocking of Fish

Guppy fries (*P. reticulata*) were purchased from a local aquarium shop and brought to the lab by using aerated plastic bags. Then the plastic bags were kept in the previously prepared (filtered, aerated and UV treated for 2 days) conditioning tank for three hours. Afterwards, the fries were gently released from the bag in the tank water and kept there for three days to being acclimatized with the laboratory conditions. Potassium permanganate (KMnO_4_) was used to disinfect the newly brought fries which reduce the mortality rate during experimental period. Fries with an average weight of 70 ± 15 mg were selected for the study. Then the fries were divided into five treatment groups, each with a triplicate replication cohort of fifteen uniformly sized individuals. Then each group were kept in a rectangular glass tank (18 × 12 × 14  inches) filled with 20 L of water maintaining the male and female ratio to 1:2 in an indoor setting.

#### 2.2.3 Feeding Experiment

Test diets were fed to the fish at 5% of their body weight twice a day in equally divided doses at six hour intervals (10:00 AM and 4:00 PM). Throughout the trial, each tank received continuous aeration to maintain the DO (mg/L) concentration at 6.5 ± 1 mg/L and pH at 7.7 to 8.4. To maintain optimum water quality, every day 1/3 of the culture water was siphoned out from the tank bottom followed by adding new water to compensate the removed amount. A complete water exchange in the tanks was done twice per week. Water temperature was maintained at 26±2 °C with a 12 h light: 12 h dark photoperiod. Total ammonia and nitrite concentrations remained below 0.02 mg. This study was carried out over 100 days to cover the growth and reproduction stages (21-day old to 120-day old) of guppy fish.

#### 2.2.4 Daily Monitoring and Record Keeping

On a daily basis, physical parameters of the culture water such as temperature, pH and DO were measured with glass thermometer, a portable pH meter (pHep-HI98107, HANNA, Romania), and dissolved oxygen meter (DO-5509, Lutron), respectively. Fish were also routinely monitored for any occurrence of stress or disease outbreak. Dead fish were removed from the aquarium as soon as they were discovered.

### 2.3 Data Collection and Analysis

#### 2.3.1 Oxidative Stress Biomarker Analysis

According to Velikova et al. (2000) [6], the level of H_2_O_2_ in fish tissues were tested on the last day of the culture period. Fresh tissue samples (0.15 g) were taken from each treatment groups for analysis. Ttrichloroacetic acid (TCA), potassium phosphate buffer (10 mM, pH 7.0) and potassium iodide were used for solution preparation. Using a spectrophotometer, the absorbance of the solution was measured at 390 nm wavelength.

Fresh fish (0.15 g) samples were prepared for the determination of lipid peroxidation level of fish tissue following Heath and Packer's method (1968) [7]. Here, the absorbance of sample solution was measured at 532 nm and 600 nm wavelength in the spectrophotometer.

Lipid peroxidation level was determined as equivalent to Malondialdehyde (MDA) and calculated as follows:

MDA equivalent (nmolg^-1^FW)=A532-A600/15,5000×10^6^

#### 2.3.2 Chemical Analysis of Water Quality

Total ammonia nitrogen (TAN), Nitrite-nitrogen (NO_2__—_N) and soluble reactive phosphorous (SRP) were determined according to the methods of Parsons et al. (1984) [8]. Water samples from different culture tanks were taken in each alternative day in a week from “day 0″ to ‘day 6″. Three different standard solutions were prepared for analysis. The optical absorbance of the prepared solutions were measured at a wavelength of 640, 543 and 885 nm by using the spectrophotometer (T80 UV/VIS Spectrophotometer) to determine the concentrations of TAN, NO_2__—_N and SRP, respectively.

#### 2.3.3 Statistical Analysis

The mean ± standard error of all the data were calculated in MS Excel and reported throughout the text. The IBM SPSS (v. 26.0) software was used to perform all statistical analyses related to the survival rate, growth characteristics, proximate composition, pigments, and oxidative stress. Descriptive statistics were computed for each treatment, followed by a test for homogeneity of variance. A one-way analysis of variance was performed to examine the acquired data (ANOVA). Tukey HSD multiple comparison tests were used to look for significant differences among treatments at 95% confidence interval level. To distinguish between groups, a post-hoc test was performed.

## Ethics Statements

These data were collected complying ARRIVE guidelines carried out in accordance with the U.K. Animals (Scientific Procedures) Act, 1986 and associated guidelines, EU Directive 2010/63/ EU for animal experiments, or the National Institutes of Health guide for the care and use of Laboratory animals (NIH Publications No. 8023, revised 1978).

## CRediT authorship contribution statement

**Razia Sultana:** Methodology, Data curation, Software, Formal analysis, Writing – original draft, Visualization. **Helena Khatoon:** Conceptualization, Project administration, Supervision. **Mohammad Redwanur Rahman:** Writing – review & editing. **Mohammad Ekramul Haque:** Data curation. **Md. Sajjadul Mustaquim:** Data curation. **Zannatul Nayma:** Software, Formal analysis. **Fardous Ara Mukta:** Software, Formal analysis.

## Declaration of Competing Interest

The authors declare that they have no known competing financial interests or personal relationships that could have appeared to influence the work reported in this paper.

## Data Availability

A comprehensive dataset on the effects of Nannochloropsis sp. inclusion diets on the growth, proximate composition, water quality, antioxidants and carotenoids concentration of guppy (Poecilia reticul (Original data) (Mendeley Data). A comprehensive dataset on the effects of Nannochloropsis sp. inclusion diets on water quality and oxidative stress of guppy (Poecilia reticulata) (Original data) (Mendeley Data).
